# Physical activity and risk of colorectal cancer in men and women.

**DOI:** 10.1038/bjc.1996.218

**Published:** 1996-05

**Authors:** I. Thune, E. Lund

**Affiliations:** Institute of Community Medicine, University of Tromsø, Norway.

## Abstract

We examined the association between self-reported occupational and recreational physical activity and the subsequent risk of colorectal cancer in a population-based cohort in Norway. During a mean follow-up time of 16.3 years for males and 15.5 years for females, 236 and 99 colon cancers and 170 and 58 rectal cancers were observed in males and females, respectively, among 53,242 males and 28,274 females who attended the screening between 1972 and 1978. Physical activity at a level equivalent to walking or bicycling for at least four hours a week during leisure-time was associated with decreased risk of colon cancer among females when compared with the sedentary group (RR = 0.62, 95% CI 0.40-0.97). Reduced risk of colon cancer was particularly marked in the proximal colon (RR = 0.51, 95% CI 0.28-0.93). This effect was not observed for occupational physical activity alone, probably due to a narrow range of self-reported physical activity at work among females. However, by combining occupational and recreational physical activity we observed an inverse dose-response effect as increasing total activity significantly reduced colon cancer risk (P for trend = 0.04). Among males 45 years or older at entry to the study, an inverse dose-response effect was observed between total physical activity and colon cancer risk (P for trend = 0.04). We also found in males a stronger preventive effect for physical activity in the proximal as compared to distal colon. In addition, we found a borderline significant decrease in colon cancer risk for occupational physical activity in males 45 years or older when compared to the sedentary group (RR = 0.74, 95% CI 0.53-1.04). All results were adjusted for age, body mass index, serum cholesterol and geographic region. No association between physical activity and rectal cancer was observed in males or females. The protective effect of physical activity on colon cancer risk is discussed in regard to energy balance, dietary factors, age, social class, body mass index and gastrointestinal transit time.


British Journal of Cancer (1996) 73, 1134-1140
© 1996 Stockton Press All rights reserved 0007-0920/96 $12.00

### Physical activity and risk of colorectal cancer in men and women

I Thune and E Lund

*Institute of Community Medicine, University of Tromsø, N-9037 Tromsø, Norway.*

**Summary** We examined the association between self-reported occupational and recreational physical activity and the subsequent risk of colorectal cancer in a population-based cohort in Norway. During a mean follow-up time of 16.3 years for males and 15.5 years for females, 236 and 99 colon cancers and 170 and 58 rectal cancers were observed in males and females, respectively, among 53 242 males and 28 274 females who attended the screening between 1972 and 1978. Physical activity at a level equivalent to walking or bicycling for at least four hours a week during leisure-time was associated with decreased risk of colon cancer among females when compared with the sedentary group (RR=0.62, 95% CI 0.40-0.97). Reduced risk of colon cancer was particularly marked in the proximal colon (RR=0.51, 95% CI 0.28-0.93). This effect was not observed for occupational physical activity alone, probably due to a narrow range of self-reported physical activity at work among females. However, by combining occupational and recreational physical activity we observed an inverse dose-response effect as increasing total activity significantly reduced colon cancer risk (*P* for trend=0.04). Among males 45 years or older at entry to the study, an inverse dose-response effect was observed between total physical activity and colon cancer risk (*P* for trend=0.04). We also found in males a stronger preventive effect for physical activity in the proximal as compared to distal colon. In addition, we found a borderline significant decrease in colon cancer risk for occupational physical activity in males 45 years or older when compared to the sedentary group (RR=0.74, 95% CI 0.53-1.04). All results were adjusted for age, body mass index, serum cholesterol and geographic region. No association between physical activity and rectal cancer was observed in males or females. The protective effect of physical activity on colon cancer risk is discussed in regard to energy balance, dietary factors, age, social class, body mass index and gastrointestinal transit time.

**Keywords:** physical activity; colorectal cancer; cohort study; gender differences; subsites

Cancer of the large intestine is one of the most common neoplasms in western countries (Muir *et al.*, 1987; Engeland *et al.*, 1993). Recently, the role of exercise in the aetiology of colon carcinogenesis has drawn particular interest. A growing number of epidemiological studies have reported a protective effect of occupational physical activity on colon cancer risk (Garabrant *et al.*, 1984; Gerhardsson *et al.*, 1986; Brownson *et al.*, 1989; Peters *et al.*, 1989; Arbman *et al.*, 1993; Chow *et al.*, 1993; Fraser and Pearce, 1993). Others have observed that recreational physical activity protects against colon cancer (Wu *et al.*, 1987; Slattery *et al.*, 1988; Gerhardsson *et al.*, 1988; Severson *et al.*, 1989; Ballard-Barbash *et al.*, 1990; Lee *et al.*, 1991; Markowitz *et al.*, 1992; Giovannuci *et al.*, 1995). In contrast, the association between physical activity and risk of rectal cancer is more inconsistent (Vena *et al.*, 1985; Gerhardsson *et al.*, 1986; Fraser and Pearce, 1993).

However, few studies have analysed the association between physical activity and colon cancer risk in females or have taken gender differences, age and subsites into consideration. In addition, patients with proximal colon cancer are older than patients with distal colon and rectal cancer, and women make up a higher percentage of patients with cancer in the proximal colon (Møller Jensen, 1984; Halvorsen, 1986; Fleshner *et al.*, 1989). Furthermore, physiological differences in the proximal and the distal colon may reflect different susceptibility to neoplastic transformation (Bufill, 1990; Dubrow *et al.*, 1993).

We therefore investigated the association between self-reported physical activity both during leisure and work and the subsequent risk of colorectal cancer in a population-based, prospective study among both sexes. We further examined whether physical activity had a different effect according to age-, gender- and site-specific colorectal cancer risk.

Correspondence: I Thune
Received 4 April 1995; revised 9 November 1995; accepted 9 November 1995

### Material and methods

Between 1972 and 1978, 104 485 males and females from five geographical areas in Norway-Oslo, Oppland, Sogn and Fjordane, Tromsø and Finnmark-were invited to participate in a population-based health survey of risk factors for cardiovascular disease. In Tromsø, all men aged 20-49 years were invited, while in Oslo men aged 40-49 were invited plus a 7% random sample of men aged 20-39. In the three counties of Oppland, Sogn and Fjordane and Finnmark all men and women aged 35-49 and a 10% random sample of persons aged 20-34 years were invited. In four small municipalities in Finnmark all men and women aged 20-34 were invited: a total of 104 485, of whom 53 622 males (73.5%) and 28 621 females (90.7%) attended the screening.

The screening procedures were similar in the five areas. Each person was invited by mail, with a covering letter and one-page questionnaire enclosed. The participants were asked to answer the questionnaire at home and bring it to the clinical examination. The clinical examination consisted of checking the questionnaire for inconsistency, measurements of weight, height and blood pressure, and the collection of blood samples. Heart rate and other measures of physical fitness were not assessed.

The questionnaire covered the following; physical activity (PhA) during recreational (R) and occupational (O) hours in the last year; history of chronic diseases especially cardiovascular symptoms and diseases, smoking habits and stress in daily life.

Self-reported physical activity categories during recreational hours were graded from 1 to 4 according to which of the following categories best described the participant's usual level of physical activity: R1=reading, watching TV or other sedentary activities; R2=walking, bicycling or physical activities for at least four hours a week; R3=exercise to keep fit, participating in recreational athletics etc. for at least four hours a week; R4=regular hard training or participation in competitive sports several times a week.

Self-reported physical activity during occupational hours was divided into four categories; O1 = mostly sedentary work; O2 = work with much walking; O3 = work with much lifting and walking; O4 = heavy manual work.

The national 11-digit personal identification number enabled a linkage to the Cancer Registry of Norway. This allowed for identification of every incident case of colorectal cancer that occurred in the cohort from the time of examination until the end of follow-up (31 December 1991). Colorectal cancers were coded according to ICD7. In some analyses, cancers in the colon were categorised as occurring in the proximal colon (153.0 + 153.1), or the distal colon (153.2 + 153.3 + 153.4). Cases identified only incidentally at autopsy were not included. Histological confirmation was obtained in 95% of the cases and among these 96.7% were adenocarcinomas and eight cases (2.5%) were classified as malignant carcinoid tumours.

In addition all 53 622 men and 28 621 women were followed up through the Norwegian Central Bureau of Statistics to identify deaths in the cohort up to the end of 1991. Those who emigrated or had a pre-existing malignancy or developed a malignancy within the first year of the study (males, $n = 380$; females, $n = 347$) were excluded from the analyses. This reduced the possibility for any undiagnosed cancer to influence the level of physical activity. The present cohort study is restricted to males and females aged 20–69 years in the follow-up period. Included for analysis were 53 242 males (867 822 person–years) and 28 274 females (437 785 person–years).

Cox's proportional hazards regression techniques were used to analyse the simultaneous effects of physical activity and possible confounders on colon and rectal cancer incidence in the cohort. In these analyses, the categories R3 and R4 of recreational physical activity were merged due to small numbers in category R4 (males, $n = 316$; females, $n = 62$). Observation years at risk of developing colon or rectal cancer were calculated as the number of years from 1 year after study entry until the time of withdrawal (year of diagnosis of cancer, time of death or end of follow-up in December 1991, which ever was earliest). In the sex-specific analyses, we adjusted for attained age (continuous variable), geographical regions and obesity at time of measurements. As a measure of obesity, we used the body mass index (BMI) (weight height$^{-2}$).

To study the influence of total physical activity on colon cancer risk, occupational (O) and recreational (R) physical activity were combined. As a reference group (R1/O1 + O2), we used sedentary leisure (R1) and both sedentary (O1) and moderate (O2) activities at work in order to increase the number of persons in the reference group.

We examined models stratified by age at entry (<45 years, ⩾45 years) and BMI (median split and tertiles) to analyse if there was any effect modification by age and BMI. Other cut-off points for age were considered without extended information. These analyses were performed with the Proc Phreg procedure in the SAS statistical package (SAS Institute, 1992). Owing to missing data, the number of subjects included in the individual analyses varies slightly.

### Results

A total of 236 colon and 170 rectal cancers among males and 99 colon and 58 rectal cancers among females were diagnosed in the study population during a mean follow-up time of 16.3 years and 15.5 years in males and females respectively. Median age at diagnosis for colon cancer was 58.1 years in males and 54.6 years in females. For rectal cancer the median age at diagnosis was 57.3 years and 55.4 years in males and females respectively. Of all cases of colorectal cancer, cases of proximal colon cancer were less frequent among males (23.4%) than among females (30.5%), whereas the proportion of distal colon cancer was reversed between the two sexes (31.8% *vs* 28.7%).

The grade of physical activity was differently distributed in males and females. Two-thirds of the females and 76% of the housewives reported frequent walking (O2) during occupational hours in contrast to only one-quarter among males (Table I). Fewer females than males reported sedentary work (O1). Gender differences were also observed during leisure time as only 10% of females reported regular training (R3 + R4) in contrast to 25.4% of males.

Age at entry was a significant risk factor in univariate analyses for both colon and rectal cancer in both sexes (Table II). A positive association was observed between body mass index (BMI) and colon cancer risk in males, but not in females. None of the variables in Table II significantly deviated from linearity when a second-order term was introduced (results not shown).

Total physical activity (occupational and recreational combined) showed an overall negative dose-response relationship with colon cancer risk among females ($P$ for trend = 0.04), but not in males (Table III).

We analysed colon cancer risk in relation to a possible age effect of total physical activity by dividing the sex-specific cohort into those younger and older than 45 years at study-entry. Among males 45 years or older at study entry (median age at diagnosis = 60.0 years), we observed a negative dose-response relationship between total physical activity and colon cancer risk ($P$ for trend = 0.04), which was not observed among males younger than 45 years at study entry (median age at diagnosis = 52.1 years) (Table IV). In addition, a borderline significant reduction on total colon cancer risk was observed among occupationally physically active males (O2, O3, O4) 45 years or older at study entry compared with the sedentary ones (RR = 0.74, 95% CI 0.53–1.04) (results not shown in Table IV). No similar age effect was observed in females.

**Table I** Self-reported physical activity during occupational (O) and recreational (R) hours among males and females aged 20–49 years at study entry

| | *Males* | | *Females* | | | | | |
|---|---|---|---|---|---|---|---|---|
| | | | *Total* | | *Housewives* | | *Non-housewives* | |
| *Physical activity (PhA)* | *Number* | *%* | *Number* | *%* | *Number* | *%* | *Number* | *%* |
| Occupational PhA | | | | | | | | |
| Sedentary (O1) | 18 737 | 35.4 | 3 232 | 11.5 | 690 | 3.4 | 2 542 | 31.4 |
| Walking (O2) | 13 990 | 26.4 | 19 192 | 68.2 | 15 221 | 76.0 | 3 971 | 49.0 |
| Lifting and walking (O3) | 11 804 | 22.3 | 4 462 | 15.9 | 3 049 | 15.2 | 1 413 | 17.4 |
| Heavy manual (O4) | 8 414 | 15.9 | 1 237 | 4.4 | 1 065 | 5.3 | 172 | 2.1 |
| Recreational PhA | | | | | | | | |
| Sedentary (R1) | 10 640 | 20.0 | 6 336 | 22.4 | 4 625 | 23.0 | 1 711 | 21.1 |
| Moderately active (R2) | 29 040 | 54.6 | 19 100 | 67.6 | 13 453 | 66.8 | 5 647 | 69.6 |
| Regular training (R3) | 12 206 | 22.9 | 2 757 | 9.8 | 2 033 | 10.1 | 724 | 8.9 |
| Regular hard training (R4) | 1 316 | 2.5 | 62 | 0.2 | 31 | 0.2 | 31 | 0.4 |

**Table II** Age-adjusted relative risk (RR) of colorectal cancer with 95% confidence interval (CI) in relation to possible risk factors. Cox's proportional hazards model

| | Colon cancer | | | | | | Rectal cancer | | | | | |
|---|---|---|---|---|---|---|---|---|---|---|---|---|
| | Males | | | Females | | | Males | | | Females | | |
| *Variable* | *No. of cases* | *RR*[a] | *95% CI* | *No. of cases* | *RR*[a] | *95% CI* | *No. of cases* | *RR*[a] | *95% CI* | *No. of cases* | *RR*[a] | *95% CI* |
| Age at entry (year) | 236 | 1.13 | (1.10–1.16) | 99 | 1.11 | (1.07–1.15) | 170 | 1.11 | (1.08–1.15) | 58 | 1.20 | (1.12–1.28) |
| BMI (1 g $cm^{-2}$) | 230 | 1.25 | (1.01–1.53) | 99 | 0.93 | (0.57–1.52) | 169 | 0.99 | (0.60–1.63) | 55 | 0.96 | (0.51–1.82) |
| Cholesterol (1 mmol $l^{-1}$) | 236 | 1.02 | (0.93–1.12) | 99 | 0.96 | (0.82–1.11) | 170 | 1.00 | (0.89–1.12) | 58 | 1.04 | (0.87–1.26) |
| Triglycerides (1 mmol $l^{-1}$) | 236 | 1.03 | (0.96–1.10) | 99 | 0.85 | (0.67–1.07) | 170 | 1.01 | (0.92–1.11) | 58 | 1.07 | (0.86–1.33) |
| Height (10 cm) | 224 | 1.13 | (0.94–1.35) | 99 | 1.08 | (0.80–1.47) | 169 | 1.02 | (0.82–1.25) | 55 | 0.88 | (0.59–1.32) |
| Smoking (10 cigarettes) | 229 | 0.96 | (0.80–1.16) | 99 | 1.11 | (0.73–1.69) | 164 | 1.06 | (0.86–1.30) | 58 | 0.84 | (0.44–1.61) |
| Married/separated (ever *vs* never) | 236 | 1.24 | (0.80–1.91) | 99 | 1.29 | (0.52–3.16) | 170 | 2.83 | (1.39–5.78) | 58 | 1.21 | (0.38–3.88) |

[a] Adjusted for age at entry. Number of cases varied slightly due to missing values for some variables.

**Table III** Adjusted relative risk (RR) of colorectal cancer with 95% confidence interval (CI) according to total physical activity (occupational (O) and recreational (R) combined) among males and females; Cox's proportional hazards model

| | Total colon cancer | | | | Proximal colon cancer | | | | Distal colon cancer | | | | Rectal cancer | | | |
|---|---|---|---|---|---|---|---|---|---|---|---|---|---|---|---|---|
| *Total Physical activity* | *No. of cases* | *RR*[a] | *95% CI* | *Trend test P value* | *No. of cases* | *RR*[a] | *95% CI* | *Trend test P value* | *No. of cases* | *RR*[a] | *95% CI* | *Trend test P value* | *No. of cases* | *RR*[a] | *95% CI* | *Trend test P value* |
| Males | | | | | | | | | | | | | | | | |
| Sedentary[b] | 26 | 1.00 | | | 10 | 1.00 | | | 14 | 1.00 | | | 18 | 1.00 | | |
| Moderate[c] | 95 | 1.18 | (0.76–1.82) | | 36 | 1.16 | (0.57–2.34) | | 56 | 1.29 | (0.72–2.33) | | 69 | 1.24 | (0.73–2.08) | |
| Active[d] | 107 | 0.97 | (0.63–1.50) | 0.49 | 48 | 0.96 | (0.47–1.93) | 0.64 | 57 | 0.99 | (0.55–1.80) | 0.53 | 81 | 1.20 | (0.72–2.02) | 0.63 |
| Females | | | | | | | | | | | | | | | | |
| Sedentary[b] | 22 | 1.00 | | | 10 | 1.00 | | | 11 | 1.00 | | | 8 | 1.00 | | |
| Moderate[c] | 17 | 0.97 | (0.33–2.77) | | 10 | 1.22 | (0.51–2.94) | | 7 | 0.84 | (0.32–2.17) | | 6 | 0.96 | (0.33–2.77) | |
| Active[d] | 59 | 0.63 | (0.39–1.04) | 0.04 | 27 | 0.62 | (0.30–1.28) | 0.10 | 27 | 0.61 | (0.30–1.23) | 0.15 | 41 | 1.27 | (0.59–2.72) | 0.45 |

[a] Adjusted for age at entry, geographic region and body mass index (BMI). [b] Sedentary (R1 + O1–2). [c] Moderate (R1 + O3–4, O1 + R3–4). [d] Active (O2–4 + R2–4).

When occupational activity was examined separately, each sex showed a consistent negative adjusted reduced risk for colon cancer in the active groups compared with the sedentary group, but in neither case was this significant (Table V). Recreational activity showed no consistent reduced trend in either sex, but females with moderate recreational physical activity (R2) had an almost 40% significant reduction in the risk of total colon cancer (RR=0.62, 95% CI 0.40–0.97). No consistent associations were observed between total physical activity, occupational or recreational physical activity and risk of rectal cancer in males or females (Table III and V).

When taking subsite into consideration, we performed site-specific analyses of the relationship between total physical activity and proximal and distal colon cancer. A negative trend for both proximal (*P* for trend = 0.10) and distal cancers (*P* for trend = 0.15) was observed in females, though this was not significant (Table III). A negative trend was observed only for proximal cancers in males older than 45 years at entry (*P* for trend = 0.08) (results not presented in Table). Further the reduction of colon cancer risk among the recreational physically active females was particularly marked in the proximal colon (RR=0.51, 95% CI 0.28–0.93) (Table VI). No corresponding subsite differences were observed in males when taking only recreational activity into consideration.

Further, we examined models stratified by BMI (median split) to analyse if there was any effect modification related to body weight (Table VI). Among females an inverse recreational physical activity–colon cancer association was stronger among leaner females (RR = 0.45, 95% CI 0.25–0.82) compared with more obese females. Among males an inverse physical activity–colon cancer association was strongest in older and leaner males. This was observed especially by dividing BMI into tertiles, as occupationally active males 45 years or older belonging to the lowest tertile (BMI < 2.33 g $cm^{-2}$) had the greatest reduction in total colon cancer risk (RR=0.50, 95% CI 0.26–0.97) (results not shown in Table VI).

To examine if the effect of physical activity on colorectal cancer differed between males and females we performed combined analyses both for total colon and for subsites. Here, we observed no significant effect of gender alone or when introducing an interaction term of gender and physical activity on colorectal cancer risk in any of the analyses (results not shown).

### Discussion

In the present study an inverse dose–response relationship between total physical activity and colon cancer risk was observed in females. In males this inverse dose–response relationship was found only for those 45 years or older at study entry. An almost 40% reduction in risk of colon cancer among the moderately leisure time active compared with sedentary females was demonstrated. This reduction in cancer risk in females from recreational physical activity was particularly related to proximal colon with an almost 50%

**Table IV** Adjusted relative risk (RR) of colon cancer with 95% confidence interval (CI) according to total physical activity (occupational (O) and recreational (R) combined) stratified by age at entry among males and females; Cox's proportional hazards model

| *Total Physical activity* | *No. of cases* | *Males RR[a]* | *Males 95% CI* | *Trends test P value* | *No. of cases* | *Females RR[a]* | *Females 95% CI* | *Trend test P value* |
|---|---|---|---|---|---|---|---|---|
| <45 years at entry | | | | | | | | |
| Sedentary[b] | 5 | 1.00 | | | 11 | 1.00 | | |
| Moderate[c] | 30 | 2.02 | (0.78–5.21) | | 8 | 0.96 | (0.39–2.40) | |
| Active[d] | 49 | 2.23 | (0.88–5.66) | 0.13 | 30 | 0.62 | (0.31–1.23) | 0.13 |
| ⩾45 years at entry | | | | | | | | |
| Sedentary[b] | 21 | 1.00 | | | 11 | 1.00 | | |
| Moderate[c] | 65 | 0.96 | (0.59–1.58) | | 9 | 0.99 | (0.41–2.39) | |
| Active[d] | 58 | 0.66 | (0.40–1.10) | 0.04 | 29 | 0.66 | (0.33–1.33) | 0.19 |

[a] Adjusted for age at entry, geographic region and body mass index (BMI). [b] Sedentary (R1 + O1–2). [c] Moderate (R1 + O3–4, O1 + R3–4). [d] Active (O2–4 + R2–4).

**Table V** Adjusted relative risk (RR) of colorectal cancer with 95% confidence interval (CI) related to categories of occupational (O) and recreational (R) physical activity among males and females; Cox's proportional hazards model

| *Physical activity (PhA)* | | *Colon cancer No. of cases* | *RR[a]* | *95% CI* | *Trend test P value* | *Rectal cancer No. of cases* | *RR[b]* | *95% CI* | *Trend test P value* |
|---|---|---|---|---|---|---|---|---|---|
| Males | | | | | | | | | |
| Occupational PhA | | | | | | | | | |
| Sedentary | (O1) | 92 | 1.00 | | | 71 | 1.00 | | |
| Walking | (O2) | 62 | 0.92 | (0.67–1.28) | | 43 | 0.90 | (0.61–1.31) | |
| Lifting/Heavy manual | (O3 + O4) | 74 | 0.82 | (0.59–1.13) | 0.22 | 54 | 1.00 | (0.69–1.45) | 0.95 |
| Recreational PhA | | | | | | | | | |
| Sedentary | (R1) | 41 | 1.00 | | | 29 | 1.00 | | |
| Moderately active | (R2) | 125 | 1.05 | (0.74–1.50) | | 106 | 1.25 | (0.83–1.89) | |
| Regular training | (R3 + R4) | 64 | 1.33 | (0.90–1.98) | 0.13 | 34 | 0.98 | (0.60–1.61) | 0.85 |
| Females | | | | | | | | | |
| Occupational PhA | | | | | | | | | |
| Sedentary | (O1) | 12 | 1.00 | | | 6 | 1.00 | | |
| Walking | (O2) | 66 | 0.82 | (0.44–1.51) | | 37 | 0.95 | (0.40–2.26) | |
| Lifting/Heavy manual | (O3 + O4) | 20 | 0.69 | (0.34–1.42) | 0.32 | 12 | 0.88 | (0.33–2.36) | 0.78 |
| Recreational PhA | | | | | | | | | |
| Sedentary | (R1) | 30 | 1.00 | | | 9 | 1.00 | | |
| Moderately active | (R2) | 57 | 0.62 | (0.40–0.97) | | 40 | 1.51 | (0.73–3.11) | |
| Regular training | (R3 + R4) | 12 | 0.84 | (0.43–1.65) | 0.25 | 6 | 1.49 | (0.53–4.22) | 0.35 |

[a] Adjusted for age at entry, geographic region and body mass index (BMI). [b] Adjusted for age at entry, geographic region, body mass index (BMI) and civil status.

**Table VI** Adjusted relative risk[a] of colon cancer with 95% confidence intervals (in parentheses) related to occupational (O) and recreational (R) physical activity stratified by subsites and body mass index (BMI) in males and females; Cox's proportional hazards model

| | No. of cases | *Occupational physical activity* Sedentary (O1) | Active (O2–4) | | No. of cases | *Recreational physical activity* Sedentary (R1) | Active (R2–4) | |
|---|---|---|---|---|---|---|---|---|
| *Males* | | | | | | | | |
| Subsites | | | | | | | | |
| Proximal | 89 | 1.00 | 0.89 | (0.57–1.18) | 90 | 1.00 | 1.05 | (0.62–1.78) |
| Distal | 127 | 1.00 | 0.82 | (0.57–1.78) | 128 | 1.00 | 1.19 | (0.75–1.89) |
| BMI (g $cm^{-2}$) | | | | | | | | |
| <2.44 | 89 | 1.00 | 0.87 | (0.56–1.35) | 89 | 1.00 | 1.36 | (0.74–2.51) |
| ⩾2.44 | 139 | 1.00 | 0.85 | (0.60–1.21) | 141 | 1.00 | 1.05 | (0.69–1.58) |
| *Females* | | | | | | | | |
| Subsites | | | | | | | | |
| Proximal | 47 | 1.00 | 1.14 | (0.41–3.18) | 48 | 1.00 | 0.51 | (0.28–0.93) |
| Distal | 45 | 1.00 | 0.52 | (0.24–1.11) | 45 | 1.00 | 0.80 | (0.41–1.56) |
| BMI (g $cm^{-2}$) | | | | | | | | |
| <2.36 | 48 | 1.00 | 1.43 | (0.51–3.98) | 48 | 1.00 | 0.45 | (0.25–0.82) |
| ⩾2.36 | 50 | 1.00 | 0.50 | (0.23–1.06) | 51 | 1.00 | 0.93 | (0.49–1.74) |

[a] Adjusted for age at entry, geographic region and body mass index (BMI).

reduction among active females. No association between physical activity and rectal cancer was observed in males or females.

The strength of this study beyond its prospective design, large size, broad population base and inclusion of both sexes, is a nearly complete cancer case ascertainment. Compulsory reporting by hospital departments and pathological laboratories for all new cases of cancer in Norway as well as death certificates results in very high case ascertainment. This is in addition to an almost 100% histological verification of colon cancer cases.

The accuracy of the self-reported physical activity questions used in the present analysis has been validated in several studies (Wilhelmsen *et al.*, 1976; Bjartveit *et al.*, 1981; Holme *et al.*, 1981; Løchen and Rasmussen 1992). Løchen and Rasmussen (1992) demonstrated that physical fitness among males increased with physical activity in leisure time. However, there are some limitations in using a single brief questionnaire reporting physical activity during one year without repeated assessments of physical activity and measurements of energy expenditure or dietary information. The large proportion (70%) of housewives in our cohort may have limited our ability to detect any effect of occupational activity on colon cancer risk among females. A greater variability in physical activity during leisure time rather than at work may in part explain why leisure time activity in females significantly reduced risk of colon cancer and occupational activity did not. In addition, the participants had to choose between only four occupational categories and four recreational levels of physical activity and we may therefore have underestimated the strength of physical activity for those most active.

The present findings support and extend previous results showing that physical activity is inversely related to colon cancer risk in humans (Garabrant *et al.*, 1984; Gerhardsson *et al.*, 1986; Brownson *et al.*, 1989; Peters *et al.*, 1989; Arbman *et al.*, 1993; Chow *et al.*, 1993; Fraser and Pearce, 1993; Wu *et al.*, 1987; Slattery *et al.*, 1988; Gerhardsson *et al.*, 1988; Severson *et al.*, 1989; Ballard-Barbash *et al.*, 1990; Lee *et al.*, 1991; Markowitz *et al.*, 1992; Giovannuci *et al.*, 1995) and animals (Andrianpopulos *et al.*, 1987; Reddy *et al.*, 1988).

We did not find an overall protective effect of total physical activity on colon cancer in males. This may be owing to the young age at entry and the fact that the number of cases of colon cancer are relatively small among the youngest males, thereby limiting the statistical power. The observation that only males 45 years or older at study entry had a protective effect of physical activity on colon cancer risk is consistent with similar findings in previous studies which support that age may be an effect modifier for colon cancer (Albanes *et al.*, 1989; Ballard-Barbash *et al.*, 1990; Slattery *et al.*, 1994). The observed 50% reduction in colon cancer risk among occupationally active, older and leaner males compared with sedentary males is in agreement with findings in the Framingham study in which the strongest inverse physical activity –large bowel cancer association was found among older (>50 years) and leaner males (Ballard-Barbash *et al.*, 1990). In contrast, no such age effect was found among females in the present nor in the Framingham study. An interpretation may be a somewhat different age distribution at diagnosis in females relative to males (median age at diagnosis; males, 58.1 years; females 54.6 years). Power may also be greater for males owing to the much greater number of cancer cases compared with females in both studies, thus making any age effect easier to discover in males. Consequently, physical activity as a protective factor in colon cancer risk may be of greater importance among the elderly relative to younger subjects in whom the importance of genetic predisposition may be greater. Biological mechanisms related to an age effect from physical activity on colon cancer risk have been proposed to act through improvements of the immune system among physically active elderly subjects (Shepard and Shek, 1995) or that physical activity, acting over a longer period of time in older people, is particularly important (Lee *et al.*, 1991). In spite of no significant gender differences from physical activity on colon cancer risk observed in the present study, previous studies suggest sex differences as men and women show differences under controlled experimental conditions in gastrointestinal transit time, stool bulk and bile acid production (Stephen *et al.*, 1986; Lampe *et al.*, 1993).

The inverse association between physical activity and colon cancer risk observed in the present study could be confounded. Physically active individuals may have had a diet with less saturated fat and more fibre than the inactive ones. Unfortunately, no dietary data were available for this analysis. However, other studies that examined dietary differences have concluded that physical activity and dietary factors are independent risk factors for colon cancer (Slattery *et al.*, 1988; Peters *et al.*, 1989; Gerhardsson de Verdier *et al.*, 1990; Whittemore *et al.*, 1990; Giovannucci *et al.*, 1994).

Holme *et al.* (1981), partly examining the same male cohort as followed in the present study, observed that higher social classes dominated among males who reported sedentary work, while males who were sedentary at leisure time more often represent lower social classes. Therefore, the reference group used in total physical activity reflect both high and low social classes. Social class did not influence colon cancer risk in a comparable society (Suadicani *et al.*, 1993). It is less likely, therefore, that social class explains the observed association between total physical activity and colon cancer risk.

Another observation of interest was the protective effect on proximal colon cancer incidence among physically leisure-time active females. This observation was in part also demonstrated in males 45 years or older at study entry with a particular reduction in proximal colon cancer incidence as a result of total physical activity. A possible explanation could be that exercise affects gut mobility more extensively in the proximal relative to distal colon. However, previous studies which have taken site-specific colon cancer risk into consideration have been inconsistent (Fraser and Pearce, 1993; Peters *et al.*, 1989; Gerhardsson de Verdier *et al.*, 1990; Vena *et al.*, 1985; Gerhardsson *et al.*, 1986; Brownson *et al.*, 1989).

Our study suggests that body size may modify the effect of physical activity as we observed leaner active males and females to be at a decreased colon cancer risk compared with obese subjects. This agrees with previous reports (Albanes *et al.*, 1989; Ballard-Barbash *et al.*, 1990; Giovannucci *et al.*, 1995). Body mass index (BMI) as a significant risk factor for colon cancer among males is consistent with previous studies (Marchand *et al.*, 1992; Ballard-Barbash *et al.*, 1990). The results have been more inconsistent in females (Albanes *et al.*, 1989; Ballard-Barbash *et al.*, 1990; Whittemore *et al.*, 1990). However, no direct mechanism has been suggested for the colon cancer–obesity association, but the association may indirectly be an effect of both diet and physical activity.

Several potential biological mechanisms may contribute to an observed protective effect of physical activity on colon cancer risk, including constipation which is often improved by physical activity. Walking (Holdstock *et al.*, 1970), running (Cordain *et al.*, 1986) and strength training (Koffler *et al.*, 1992), have generally been found to reduce GI transit times although not in one study (Bingham and Cummings, 1989). Contact between the colon mucosa and potential carcinogens in the faecal stream may be decreased by exercise because of shortened transit time. The fact that physical activity does not seem to lower the risk of rectal cancer accords with this 'transit time theory' as the rectum is only intermittently filled with faeces and colon peristalsis has less influence on the faecal transit time in the rectum. A decrease in the ratio of secondary to primary bile acids has been observed in obese patients after treatment with subcaloric diet and graded physical activity (Kadyrova and Shakieva, 1986). This effect of physical activity may be of importance since a high excretion of bile acids may increase the risk of colon cancer. Exercise can also elevate the production of some prostaglandins that, in turn, may influence colon cancer risk (Demers *et al.*, 1981). Physical activity may also increase colonic bloodflow so that faecal mutagens are transported away from the mucous membrane.

In conclusion, our study supports a protective effect of total physical activity on colon cancer, but not rectal cancer, in both males and females. In males this protective effect of physical activity is of greatest importance among the elderly. The stronger protective effect of physical activity on proximal rather than distal colon cancer risk supports the assumption that physical activity affects gut mobility more extensively in the proximal relative to distal colon. Further studies are needed in which repeated measurements of duration and intensity of physical activity besides energy balance, dietary factors, age, subsites and gender differences are taken into account.

**Acknowledgements**
This research is made available by those conducting the Oslo study, and by data made available by the National Health Screening Service, University of Tromsø, and the Cancer Registry of Norway. Financial support was given by the Norwegian Cancer Society.

### References

ALBANES D, BLAIR AA AND TAYLOR PR. (1989). Physical activity and risk of cancer in the NHANES I population. *Am. J. Public Health*, **79**, 744–750.

ANDRIANPOPULOS G, NELSON RL, BOMBECK CT AND SOUZA G. (1987). The influence of physical activity in 1,2-dimethylhydrazine induced colon carcinogenesis in the rat. *Anticancer Res.*, **7**, 849–852.

ARBMAN G, AXELSON O, FREDRIKSSON M, NILSSON E AND SJØDAHL R. (1993). Do occupational factors influence the risk of colon and rectal cancer in different ways? *Cancer*, **72**, 2543–2549.

BALLARD-BARBASH R, SCHATZKIN A, ALBANES D, SCHIFFMAN MH, KREGER BE, KANNEL WB, ANDERSON KM AND HELSEL WE. (1990). Physical activity and risk of large bowel cancer in the Framingham Study. *Cancer Res.*, **50**, 3610–3613.

BINGHAM SA AND CUMMINGS JH. (1989). Effect of exercise and physical fitness on large intestinal function. *Gastroenterology*, **97**, 1389–1399.

BJARTVEIT K, FOSS OP AND GJERVIG T. (1981). The cardiovascular disease study in Norwegian counties. Results from first screening. *Acta Med. Scand.*, **209**, 277–283.

BROWNSON RC, ZAHM SH, CHANG JC AND BLAIR AA. (1989). Occupational risk of colon cancer. An analysis by anatomic subsite. *Am. J. Epidemiol.*, **130**, 675–687.

BUFILL JA. (1990). Colorectal cancer: Evidence for distinct genetic categories based on proximal or distal tumor location. *Ann. Int. Med.*, **113**, 779–788.

CHOW WH, DOSEMECI M, ZHENG W, VETTER R, MCLAUGHLIN JK, GAO YT AND BLOT WJ. (1993). Physical activity and occupational risk of colon cancer in Shanghai, China. *Int. J. Epidemiol.*, **22**, 23–29.

CORDAIN L, LATIN RW AND BEHNKE JJ. (1986). The effects of an aerobic running program on bowel transit time. *J. Sports Med.*, **26**, 101–104.

DEMERS LM, HARRISON TS, HALBERT DR AND SANTEN RJ. (1981). Effect of prolonged exercise on plasma prostaglandin levels. *Prostaglandins Med.*, **6**, 413–418.

DUBROW R, BERNSTEIN J AND HOLFORD TR. (1993). Age-period-cohort modelling of large-bowel-cancer incidence by anatomic sub-site and sex in Connecticut. *Int. J. Cancer*, **53**, 907–913.

ENGELAND A, HALDORSEN T, TRETLI S, HAKULINEN T, HØRTE LG, LUOSTARINEN T, MAGNUS K, SCHAU G, SIGVALDASON H, STORM HH, TULENIUS H AND VAITTINEN P. (1993). Prediction of cancer incidence in the Nordic countries up to the years 2000 and 2010. *APMIS*, **38(suppl.)**, 101.

FLESHNER P, SLATER G AND AUFSES AH. (1989). Age and sex distribution of patients with colorectal cancer. *Dis. Colon Rectum*, **32**, 107–111.

FRASER G AND PEARCE N. (1993). Occupational physical activity and risk of cancer of the colon and the rectum in New Zealand males. *Cancer Causes Control*, **4**, 45–50.

GARABRANT DH, PETERS JM, MACK TM AND BERNSTEIN L. (1984). Job activity and colon cancer risk. *Am. J. Epidemiol.*, **119**, 1005–1014.

GERHARDSSON M, NORELL SE, KIVIRANTA H, PEDERSEN NL AND AHLBOM A. (1986). Sedentary jobs and colon cancer. *Am. J. Epidemiol.*, **123**, 775–780.

GERHARDSSON M, FLODERUS B AND NORELL SE. (1988). Physical activity and colon cancer risk. *Int. J. Epidemiol.*, **17**, 743–746.

GERHARDSSON DE VERDIER M, STEINECK G, HAGMAN U, RIEGER Å AND NORELL SE. (1990). Physical activity and colon cancer: A case-referent study in Stockholm. *Int. J. Cancer*, **46**, 985–989.

GIOVANNUCCI E, RIMM EB, STAMPFER MJ, COLDITZ GA, ASCHERIO A AND WILLETT WC. (1994). Intake of fat, meat, and fibre in relation to risk of colon cancer in men. *Cancer Res.*, **54**, 2390–2397.

GIOVANNUCCI E, ASCHERIO A, RIMM EB, COLDITZ GA, STAMPFER MJ AND WILLETT WC. (1995). Physical activity, obesity, and risk for colon cancer and adenoma in men. *Ann. Int. Med.*, **122**, 327–334.

HALVORSEN TB. (1986). Site distribution of colorectal adenocarcinomas. A rectrospective study of 853 tumours. *Scand. J. Gastroenterol.*, **21**, 973–978.

HOLDSTOCK DJ, MISIEWICZ JJ, SMITH T AND ROWLANDS EN. (1970). Propulsion (mass movements) in the human colon and its relationship to meals and somatic activity. *Gut.*, **11**, 91–99.

HOLME I, HELGELAND A, HJERMANN I, LEREN P AND LUND-LARSEN PG. (1981). Physical activity at work and at leisure in relation to cornary risk factors and social class. A 4-year mortality follow-up. The Oslo Study. *Acta Med. Scand.*, **209**, 277–283.

KADYROVA RK AND SHAKIEVA RA. (1986). Dynamics of changes in the lipid composition of bile in patients with alimentary obesity during treatment. *Ter. Arkh. (Moscow)*, **58**, 79–82.

KOFFLER KH, MENKES A, REDMOND RA, WHITEHEAD WE, PRATLEY RE AND HURLEY BF. (1992). Strength training accelerates gastrointestinal transit in middle-aged and older men. *Med. Sci. Sports and Exercise*, **24**, 415–419.

LAMPE JW, FREDSTROM SB, SLAVIN JL AND POTTER JD. (1993). Sex differences in colonic function: a randomized trial. *Gut*, **34**, 531–536.

LEE I-MIN, PAFFENBARGER JR. RS AND HSIEH CC. (1991). Physical activity and risk of developing colorectal cancer among college alumni. *J. Natl Cancer Inst.*, **83**, 1324–1329.

LØCHEN M-L AND RASMUSSEN K. (1992). The Tromsø study: physical fitness, self reported physical activity, and their relationship to other coronary risk factors. *J. Epidemiol. Commun. Health*, **26**, 103–107.

MARCHAND LL, WILKENS LR AND MI M-P. (1992). Obesity in youth and middle age and risk of colorectal cancer in men. *Cancer Causes Control*, **3**, 349–354.

MARKOWITZ S, MORABIA A, GARIBALDI K AND WYNDER E. (1992). Effect of occupational and recreational activity on the risk of colorectal cancer among males: a case-control study. *Int. J. Epidemiol.*, **21**, 1057–1062.

MØLLER JENSEN OM. (1984). Different age and sex relationship for cancer of subsites of the large bowel. *Br. J. Cancer*, **50**, 825–829.

MUIR C, WATERHOUSE J. MACK T, POWELL J AND WHELAN S. (1987). *Cancer Incidence in Five Continents*. Vol. V. IARC Scientific publication. **88.** IARC: Lyon.

PETERS RK, GARABRANT DH, YU MC AND MACK TM. (1989). A case–control study of occupational and dietary factors in colorectal cancer in young men by subsite. *Cancer Res.*, **49**, 5459–5468.

REDDY BS, SUGIE S AND LOWENFELS A. (1988). Effect of voluntary exercise on azoxymethane-induced colon carcinogenesis in male F344 rats. *Cancer Res.*, **48**, 7079–7081.

SAS INSTITUTE. (1992). *SAS/STAT Guide for Personal Computers*. Version 6 edition. SAS Institute: Cary, NC (USA).

SEVERSON RK, NOMURA AMY, GROVE JS AND STEMMERMANN GN. (1989). A prospective analysis of physical activity and cancer. *Am. J. Epidemiol.*, **130**, 522–529.

SHEPHARD RJ AND SHEK PN. (1995). Exercise, aging and immune function. *Int. J. Sports Med.*, **16**, 1–6.

SLATTERY ML, SCHUMACHER MC, SMITH KR, WEST DW AND ABD-ELGHANY N. (1988). Physical activity, diet, and risk of colon cancer in Utah. *Am. J. Epidemiol.*, **128**, 989–999.

SLATTERY ML, ABD-ELGHANY N, KERBER R AND SCHUMACHER MC. (1990). Physical activity and colon cancer: a comparison of various indicators of physical activity to evaluate the association. *Epidemiology*, **1**, 481–485.

SLATTERY ML, POTTER JD AND SORENSON AW. (1994). Age and risk factors for colon cancer (United States and Australia): are there implications for understanding differences in case control and cohort studies? *Cancer Causes Control*, **5**, 557–563.

STEPHEN AM, WIGGINS HS, ENGLYST HN, COLE TJ, WAYMAN BJ AND CUMMINGS JH. (1986). The effect of age, sex, and level of intake of dietary fibre from wheat on large-bowel function in thirty healthy subjects. *Br. J. Nutr.*, **56**, 349–361.

SUADICANI P, HEIN HO AND GYNTELBERG F. (1993). Height, weight, and risk of colorectal cancer. (1993). *Scand. J. Gastroenterol.*, **28**, 285–288.

VENA JE, GRAHAM S, ZIELEZNY M, SWANSON MK, BARNES RE AND NOLAN J. (1985). Lifetime occupational exercise and colon cancer. *Am. J. Epidemiol.*, **122**, 357–365.

WHITTEMORE AS, WU-WILLIAMS AH, LEE M, SHU Z, GALLAGHER RP, DENG-AO J, LUN Z, XIANGHUI W, KUN C, JUNG D, TEH C-Z, CHENGDE L, YAO XJ, PAFFENBARGER JR. RS AND HENDERSON BE. (1990). Diet, physical activity, and colorectal cancer among chinese in North America and China. *J. Natl. Cancer Inst.*, **82**, 915–926.

WILHELMSEN L, TIBBLIN G, AURELL M, BJURE J, EKSTRØM-JODAL B AND GRIMBY G. (1976). Physical activity, physical fitness and risk of myocardial infarction. *Adv. Cardiol.*, **18**, 217–230.

WU AH, PAGANINI-HILL A, ROSS RK AND HENDERSON BE. (1987). Alcohol, physical activity and other risk factors for colorectal cancer: a prospective study. *Br. J. Cancer.*, **55**, 687–694.


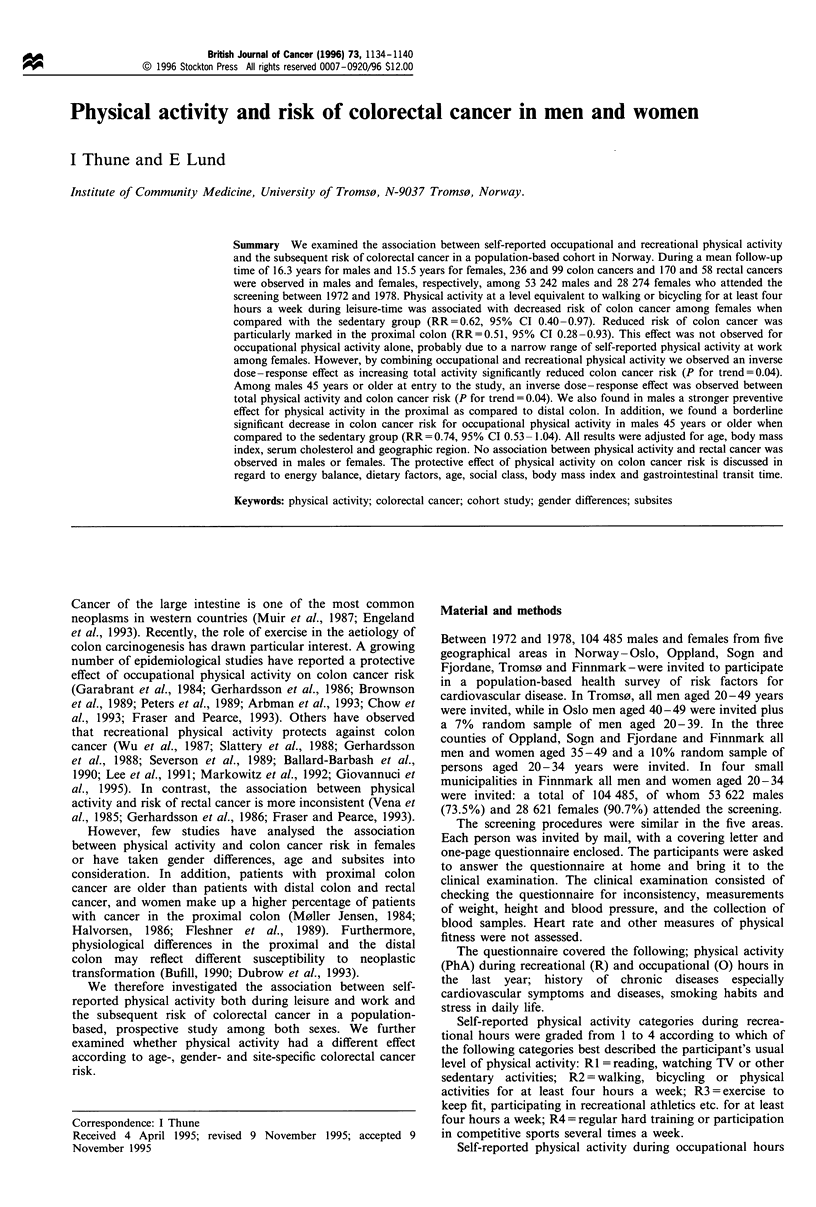

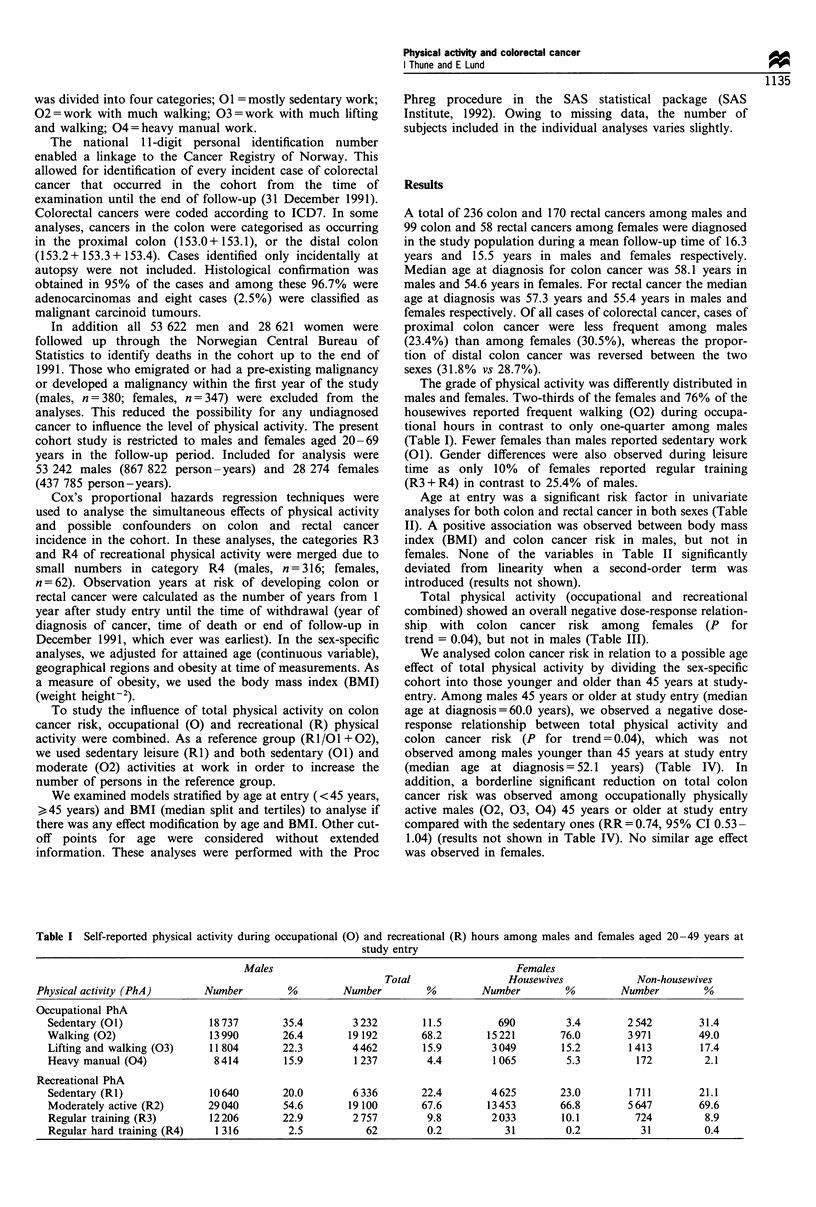

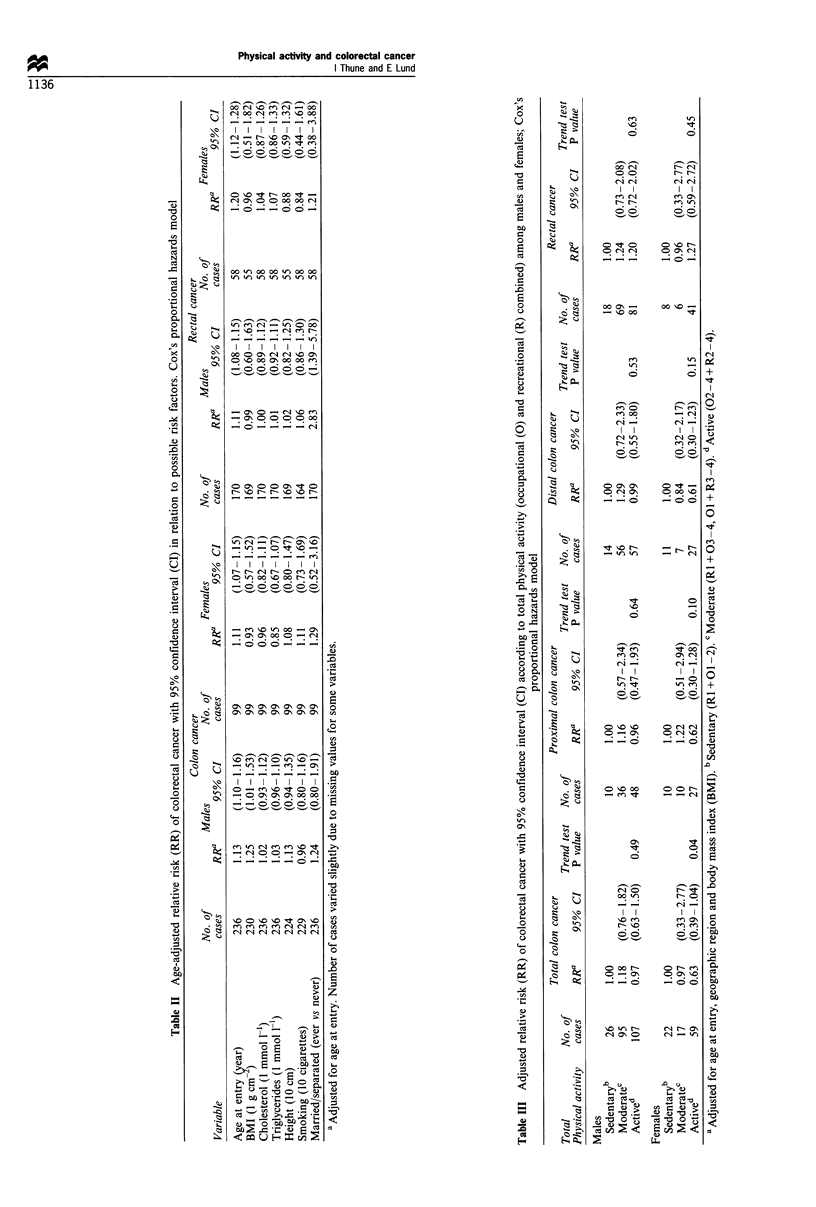

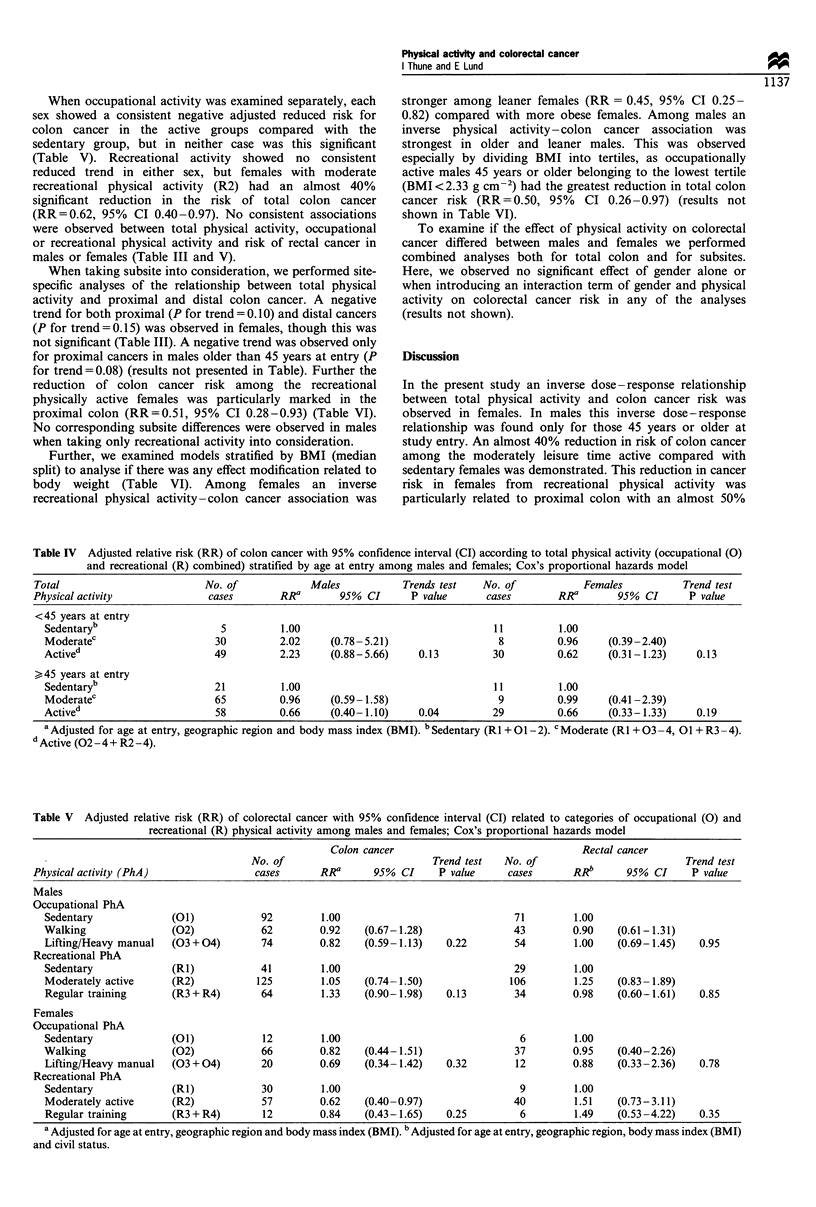

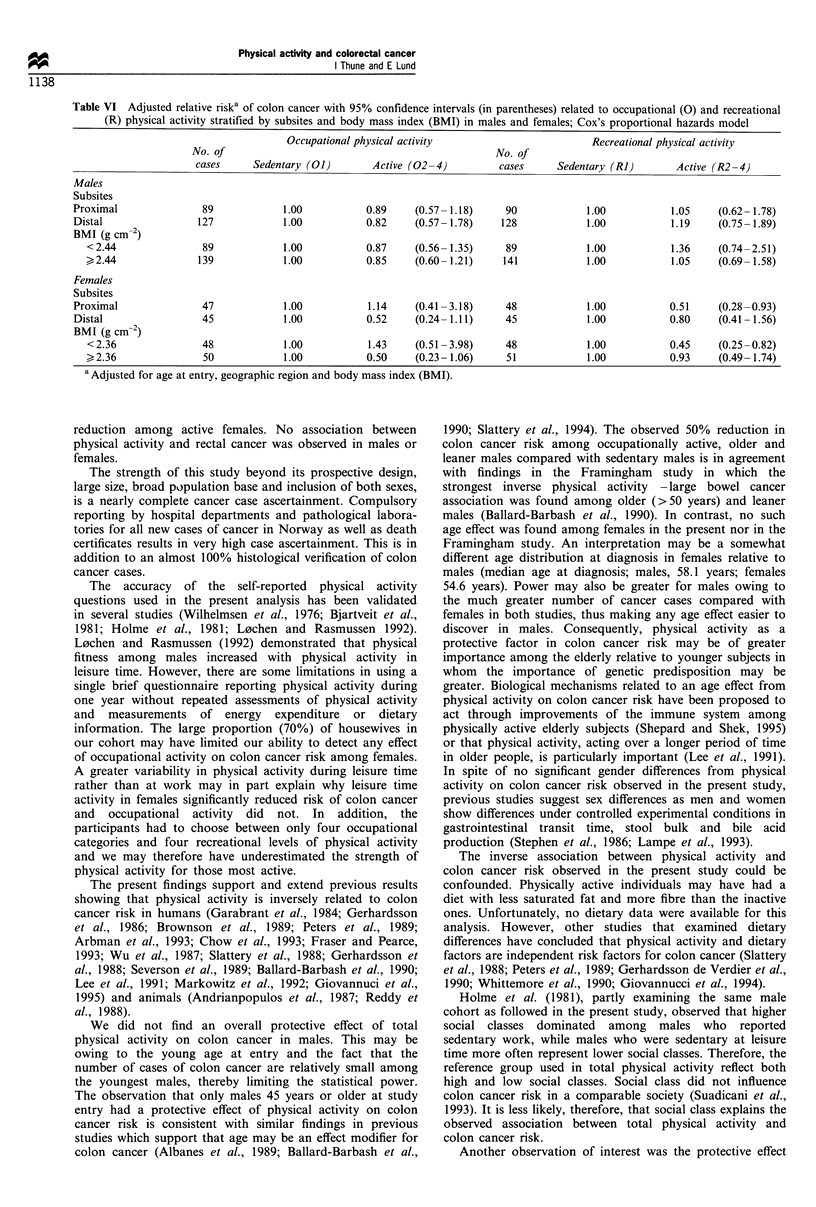

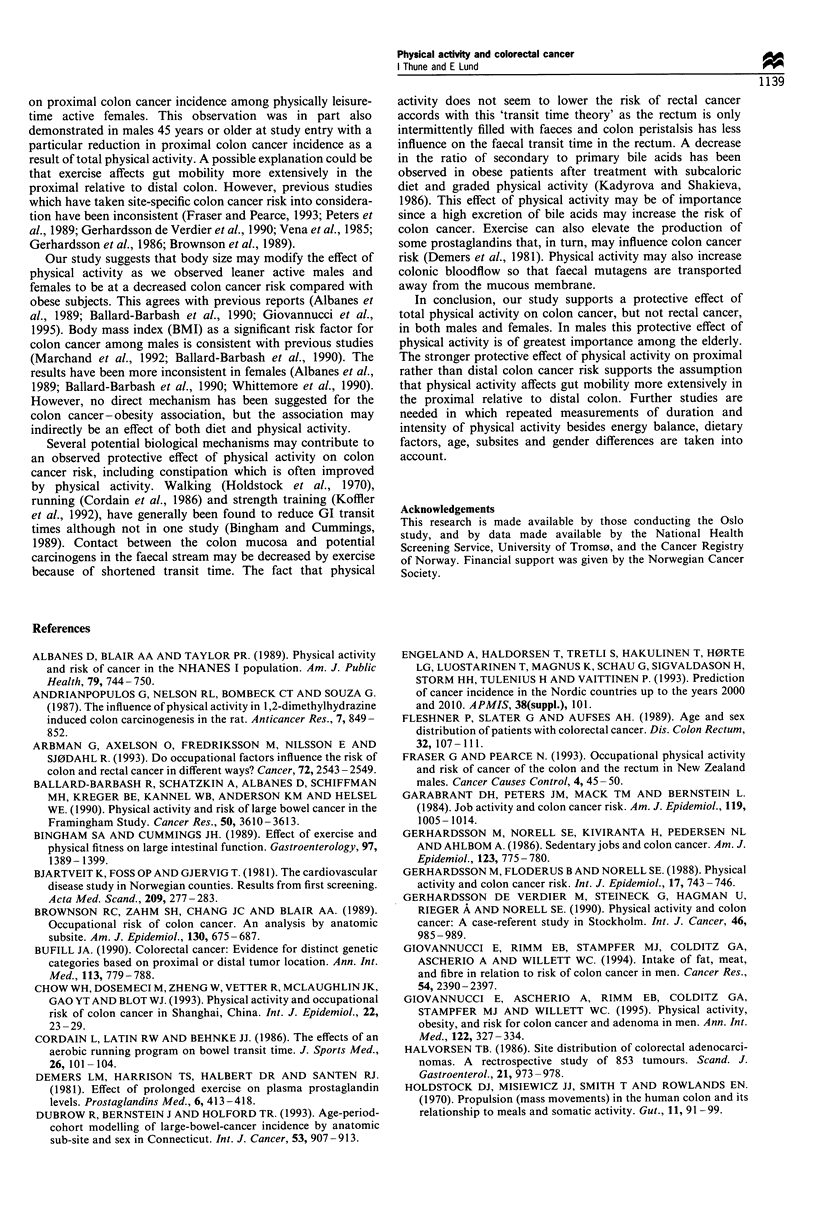

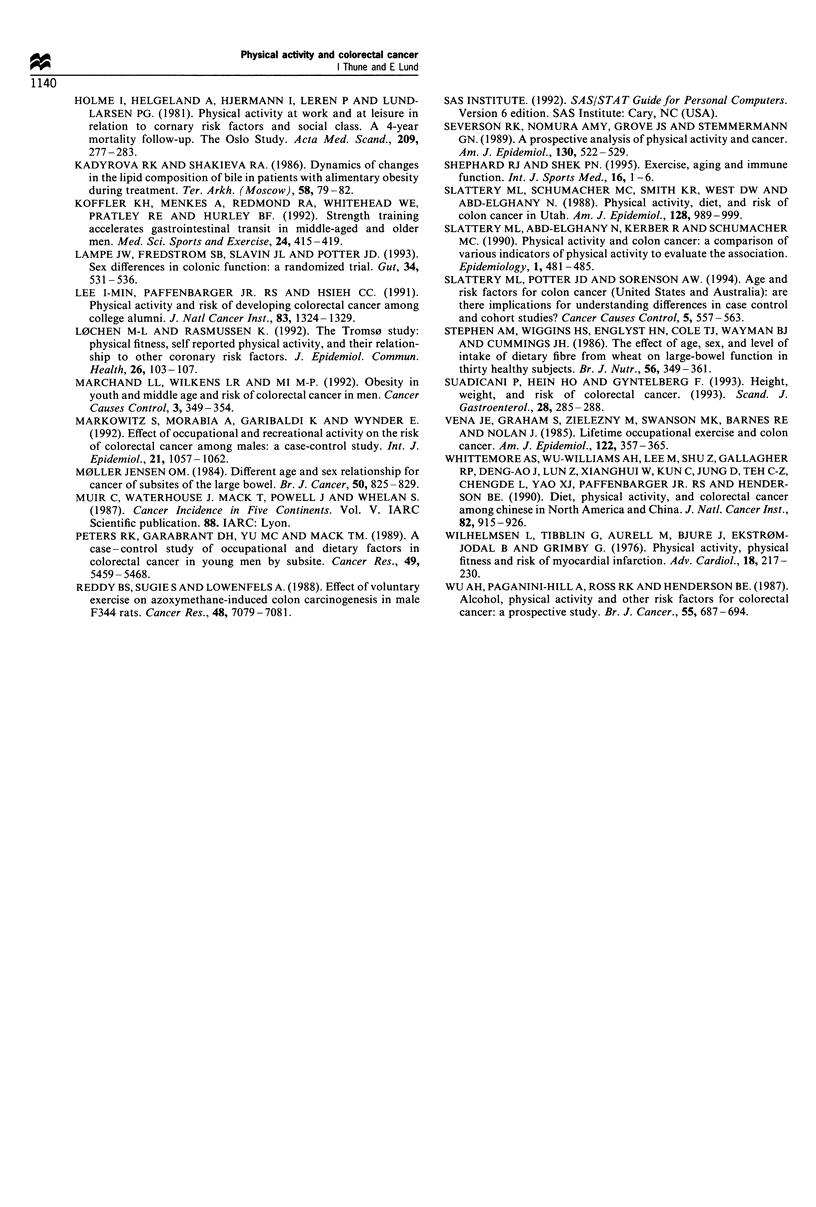


## References

[OCR_01161] Albanes D., Blair A., Taylor P. R. (1989). Physical activity and risk of cancer in the NHANES I population.. Am J Public Health.

[OCR_01166] Andrianopoulos G., Nelson R. L., Bombeck C. T., Souza G. (1987). The influence of physical activity in 1,2 dimethylhydrazine induced colon carcinogenesis in the rat.. Anticancer Res.

[OCR_01174] Arbman G., Axelson O., Fredriksson M., Nilsson E., Sjödahl R. (1993). Do occupational factors influence the risk of colon and rectal cancer in different ways?. Cancer.

[OCR_01176] Ballard-Barbash R., Schatzkin A., Albanes D., Schiffman M. H., Kreger B. E., Kannel W. B., Anderson K. M., Helsel W. E. (1990). Physical activity and risk of large bowel cancer in the Framingham Study.. Cancer Res.

[OCR_01184] Bingham S. A., Cummings J. H. (1989). Effect of exercise and physical fitness on large intestinal function.. Gastroenterology.

[OCR_01192] Brownson R. C., Zahm S. H., Chang J. C., Blair A. (1989). Occupational risk of colon cancer. An analysis by anatomic subsite.. Am J Epidemiol.

[OCR_01197] Bufill J. A. (1990). Colorectal cancer: evidence for distinct genetic categories based on proximal or distal tumor location.. Ann Intern Med.

[OCR_01202] Chow W. H., Dosemeci M., Zheng W., Vetter R., McLaughlin J. K., Gao Y. T., Blot W. J. (1993). Physical activity and occupational risk of colon cancer in Shanghai, China.. Int J Epidemiol.

[OCR_01210] Cordain L., Latin R. W., Behnke J. J. (1986). The effects of an aerobic running program on bowel transit time.. J Sports Med Phys Fitness.

[OCR_01215] Demers L. M., Harrison T. S., Halbert D. R., Santen R. J. (1981). Effect of prolonged exercise on plasma prostaglandin levels.. Prostaglandins Med.

[OCR_01220] Dubrow R., Bernstein J., Holford T. R. (1993). Age-period-cohort modelling of large-bowel-cancer incidence by anatomic sub-site and sex in Connecticut.. Int J Cancer.

[OCR_01230] Fleshner P., Slater G., Aufses A. H. (1989). Age and sex distribution of patients with colorectal cancer.. Dis Colon Rectum.

[OCR_01235] Fraser G., Pearce N. (1993). Occupational physical activity and risk of cancer of the colon and rectum in New Zealand males.. Cancer Causes Control.

[OCR_01240] Garabrant D. H., Peters J. M., Mack T. M., Bernstein L. (1984). Job activity and colon cancer risk.. Am J Epidemiol.

[OCR_01256] Gerhardsson de Verdier M., Steineck G., Hagman U., Rieger A., Norell S. E. (1990). Physical activity and colon cancer: a case-referent study in Stockholm.. Int J Cancer.

[OCR_01250] Gerhardsson M., Floderus B., Norell S. E. (1988). Physical activity and colon cancer risk.. Int J Epidemiol.

[OCR_01245] Gerhardsson M., Norell S. E., Kiviranta H., Pedersen N. L., Ahlbom A. (1986). Sedentary jobs and colon cancer.. Am J Epidemiol.

[OCR_01266] Giovannucci E., Ascherio A., Rimm E. B., Colditz G. A., Stampfer M. J., Willett W. C. (1995). Physical activity, obesity, and risk for colon cancer and adenoma in men.. Ann Intern Med.

[OCR_01263] Giovannucci E., Rimm E. B., Stampfer M. J., Colditz G. A., Ascherio A., Willett W. C. (1994). Intake of fat, meat, and fiber in relation to risk of colon cancer in men.. Cancer Res.

[OCR_01272] Halvorsen T. B. (1986). Site distribution of colorectal adenocarcinomas. A retrospective study of 853 tumours.. Scand J Gastroenterol.

[OCR_01277] Holdstock D. J., Misiewicz J. J., Smith T., Rowlands E. N. (1970). Propulsion (mass movements) in the human colon and its relationship to meals and somatic activity.. Gut.

[OCR_01288] Holme I., Helgeland A., Hjermann I., Leren P., Lund-Larsen P. G. (1981). Physical activity at work and at leisure in relation to coronary risk factors and social class. A 4-year mortality follow-up. The Oslo study.. Acta Med Scand.

[OCR_01331] Jensen O. M. (1984). Different age and sex relationship for cancer of subsites of the large bowel.. Br J Cancer.

[OCR_01298] Koffler K. H., Menkes A., Redmond R. A., Whitehead W. E., Pratley R. E., Hurley B. F. (1992). Strength training accelerates gastrointestinal transit in middle-aged and older men.. Med Sci Sports Exerc.

[OCR_01304] Lampe J. W., Fredstrom S. B., Slavin J. L., Potter J. D. (1993). Sex differences in colonic function: a randomised trial.. Gut.

[OCR_01320] Le Marchand L., Wilkens L. R., Mi M. P. (1992). Obesity in youth and middle age and risk of colorectal cancer in men.. Cancer Causes Control.

[OCR_01309] Lee I. M., Paffenbarger R. S., Hsieh C. (1991). Physical activity and risk of developing colorectal cancer among college alumni.. J Natl Cancer Inst.

[OCR_01316] Løchen M. L., Rasmussen K. (1992). The Tromsø study: physical fitness, self reported physical activity, and their relationship to other coronary risk factors.. J Epidemiol Community Health.

[OCR_01325] Markowitz S., Morabia A., Garibaldi K., Wynder E. (1992). Effect of occupational and recreational activity on the risk of colorectal cancer among males: a case-control study.. Int J Epidemiol.

[OCR_01341] Peters R. K., Garabrant D. H., Yu M. C., Mack T. M. (1989). A case-control study of occupational and dietary factors in colorectal cancer in young men by subsite.. Cancer Res.

[OCR_01345] Reddy B. S., Sugie S., Lowenfels A. (1988). Effect of voluntary exercise on azoxymethane-induced colon carcinogenesis in male F344 rats.. Cancer Res.

[OCR_01354] Severson R. K., Nomura A. M., Grove J. S., Stemmermann G. N. (1989). A prospective analysis of physical activity and cancer.. Am J Epidemiol.

[OCR_01370] Slattery M. L., Abd-Elghany N., Kerber R., Schumacher M. C. (1990). Physical activity and colon cancer: a comparison of various indicators of physical activity to evaluate the association.. Epidemiology.

[OCR_01376] Slattery M. L., Potter J. D., Sorenson A. W. (1994). Age and risk factors for colon cancer (United States and Australia): are there implications for understanding differences in case-control and cohort studies?. Cancer Causes Control.

[OCR_01366] Slattery M. L., Schumacher M. C., Smith K. R., West D. W., Abd-Elghany N. (1988). Physical activity, diet, and risk of colon cancer in Utah.. Am J Epidemiol.

[OCR_01380] Stephen A. M., Wiggins H. S., Englyst H. N., Cole T. J., Wayman B. J., Cummings J. H. (1986). The effect of age, sex and level of intake of dietary fibre from wheat on large-bowel function in thirty healthy subjects.. Br J Nutr.

[OCR_01388] Suadicani P., Hein H. O., Gyntelberg F. (1993). Height, weight, and risk of colorectal cancer. An 18-year follow-up in a cohort of 5249 men.. Scand J Gastroenterol.

[OCR_01394] Vena J. E., Graham S., Zielezny M., Swanson M. K., Barnes R. E., Nolan J. (1985). Lifetime occupational exercise and colon cancer.. Am J Epidemiol.

[OCR_01396] Whittemore A. S., Wu-Williams A. H., Lee M., Zheng S., Gallagher R. P., Jiao D. A., Zhou L., Wang X. H., Chen K., Jung D. (1990). Diet, physical activity, and colorectal cancer among Chinese in North America and China.. J Natl Cancer Inst.

[OCR_01404] Wilhelmsen L., Tibblin G., Aurell M., Bjure J., Ekström-Jodal B., Grimby G. (1976). Physical activity, physical fitness and risk of myocardial infarction.. Adv Cardiol.

[OCR_01410] Wu A. H., Paganini-Hill A., Ross R. K., Henderson B. E. (1987). Alcohol, physical activity and other risk factors for colorectal cancer: a prospective study.. Br J Cancer.

